# Supplementary material for: Structure of the native γ-tubulin ring complex capping spindle microtubules
Source: Nat Struct Mol Biol. 2024 Apr 12;31(7):1134–44. doi: 10.1038/s41594-024-01281-y (PMC11257966; doi:10.1038/s41594-024-01281-y)
Supplement: Supplementary file 1 — Reporting Summary [file 41594_2024_1281_MOESM1_ESM.pdf]

nature portfolio

Corresponding author(s): Tom Dendooven, Stanislau Yatskevich, John Kilmartin, David Barford

Last updated by author(s): Mar 6, 2024

Reporting Summary

Nature Portfolio wishes to improve the reproducibility of the work that we publish. This form provides structure for consistency and transparency in reporting. For further information on Nature Portfolio policies, see our Editorial Policies and the Editorial Policy Checklist.

Statistics

For all statistical analyses, confirm that the following items are present in the figure legend, table legend, main text, or Methods section.

n/a	Confirmed	
☒	☐	The exact sample size (*n*) for each experimental group/condition, given as a discrete number and unit of measurement
☒	☐	A statement on whether measurements were taken from distinct samples or whether the same sample was measured repeatedly
☒	☐	The statistical test(s) used AND whether they are one- or two-sided<br>*Only common tests should be described solely by name; describe more complex techniques in the Methods section.*
☒	☐	A description of all covariates tested
☒	☐	A description of any assumptions or corrections, such as tests of normality and adjustment for multiple comparisons
☒	☐	A full description of the statistical parameters including central tendency (e.g. means) or other basic estimates (e.g. regression coefficient) AND variation (e.g. standard deviation) or associated estimates of uncertainty (e.g. confidence intervals)
☒	☐	For null hypothesis testing, the test statistic (e.g. *F*, *t*, *r*) with confidence intervals, effect sizes, degrees of freedom and *P* value noted<br>*Give P values as exact values whenever suitable.*
☒	☐	For Bayesian analysis, information on the choice of priors and Markov chain Monte Carlo settings
☒	☐	For hierarchical and complex designs, identification of the appropriate level for tests and full reporting of outcomes
☒	☐	Estimates of effect sizes (e.g. Cohen's *d*, Pearson's *r*), indicating how they were calculated

Our web collection on statistics for biologists contains articles on many of the points above.

Software and code

Policy information about availability of computer code

Data collection	EM data collection free software was used: SerialEM v4.2 for automated cryo-EM data collection
Data analysis	Cryo-electron tomography:<br>Micrograph movie frames were aligned with Warp v1.09. CTF estimation was performed using Warp v1.09. Tilts series alignment and particle picking were done using Dynamo v1.1.514. Sub-tomogram averaging was done in Relion v3.0 and v3.1 and M v1.09. Models were built using Chimera X v1.4 and COOT v0.8.9.2 and real-space refinement in PHENIX v1.18.2.<br><br>Model validation was performed using PHENIX v1.18.2. Visualization was done using Chimera X v1.4 and ArtiaX v0.3.<br>Structure prediction was performed with the ALPHAFOLD2 implemented in COLABFOLD v1.5.2.<br><br>Protein crystallography: Data were processed using MOSFLM v7.4.0 and AIMLESS v0.7.4 (CCP4 v8.0). The structure was determined using Phaser v2.8.3 and refined using Phenix v1.17.1.<br><br>Crosslinking mass spectrometry (CLMS): Protein identification was carried out using MaxQuant (v2.4.10.0).<br>For size exclusion chromatography fractions, MS2 peak lists were generated using the MSConvert module in ProteoWizard (v3.0.11729), and identification of crosslinked peptides was performed using xiSEARCH software (v1.7.6.4), and crosslinks were visualised in xiView v1.0.0.<br><br>The Napari sub-boxer code is available from https://github.com/alisterburt/napari-subboxer.git.

For manuscripts utilizing custom algorithms or software that are central to the research but not yet described in published literature, software must be made available to editors and reviewers. We strongly encourage code deposition in a community repository (e.g. GitHub). See the Nature Portfolio guidelines for submitting code & software for further information.

Data

Policy information about availability of data

All manuscripts must include a data availability statement. This statement should provide the following information, where applicable:

- Accession codes, unique identifiers, or web links for publicly available datasets
- A description of any restrictions on data availability
- For clinical datasets or third party data, please ensure that the statement adheres to our policy

Atomic coordinates and cryo-EM density maps of the γTuRC capping MTs (PDBs: 8QV2, 8QV3; maps: EMD-18665, EMD-18666), and MT lattice (PDB: 8QV0 ;map: EMD-18664) have been deposited in the Protein Data Bank (www.rcsb.org) and the Electron Microscopy Data Bank (https://ebi.ac.uk/pdbe/emdb/), respectively and also listed in Table 1.

The atomic coordinates and crystallographic data for Spc98NHD (PDB: 8QRY) have been deposited in the Protein Data Bank (www.rcsb.org) and listed in Table 2.

CLMS data have been deposited in jPOST (accession code JPST002974 [PRIDE dataset identifier PXD050440]).

The mass spectrometry proteomics data have been deposited to the ProteomeXchange Consortium via the PRIDE partner repository with the dataset identifier PXD050372.

Data availability.
All data are available in the main text, Methods and Supplementary and Extended Data. Atomic coordinates and cryo-EM density maps of the ⍰TuRC capping MTs (PDBs: 8QV2, 8QV3; maps: EMD-18665, EMD-18666), and MT lattice (PDB: 8QV0 ;map: EMD-18664) have been deposited in the Protein Data Bank (www.rcsb.org) and the Electron Microscopy Data Bank (https://ebi.ac.uk/pdbe/emdb/), respectively and also listed in Table 1. The atomic coordinates and crystallographic data for Spc98NHD (PDB: 8QRY) have been deposited in the Protein Data Bank (www.rcsb.org) and listed in Table 2. CLMS data have been deposited in jPOST (accession code JPST002974 [PRIDE dataset identifier PXD050440]). The mass spectrometry proteomics data have been deposited to the ProteomeXchange Consortium via the PRIDE partner repository with the dataset identifier PXD050372.

Code availability.
Source code for the Napari sub-boxer is available from (https://github.com/alisterburt/napari-subboxer.git).

Research involving human participants, their data, or biological material

Policy information about studies with human participants or human data. See also policy information about sex, gender (identity/presentation), and sexual orientation and race, ethnicity and racism.

Reporting on sex and gender	N/A
Reporting on race, ethnicity, or other socially relevant groupings	N/A
Population characteristics	N/A
Recruitment	N/A
Ethics oversight	N/A

Note that full information on the approval of the study protocol must also be provided in the manuscript.

Field-specific reporting

Please select the one below that is the best fit for your research. If you are not sure, read the appropriate sections before making your selection.

☒ Life sciences ☐ Behavioural & social sciences ☐ Ecological, evolutionary & environmental sciences

For a reference copy of the document with all sections, see nature.com/documents/nr-reporting-summary-flat.pdf

Life sciences study design

All studies must disclose on these points even when the disclosure is negative.

Sample size	For the budding yeast spindle pole body, a total of 364 tilt series were collected. A total number of 7,910 particles were picked manually for yTuRC and used for the final reconstruction. These are typical image numbers for cryo-ET data sets and were sufficient to obtain sub-nanometer reconstructions of yTuRC
Data exclusions	3D classification procedures did not reveal broken particles or particles that do not belong to classes of interest, as all particles were picked manually for each tomogram. This is a standard practice in cryo-ET studies.

Replication	Cryo-ET datasets were collected with multiple samples in separate imaging sessions, all data collections were successful. All genetic experiments were repeated at least in three independent experiments and are all reproducible.
Randomization	For cryo-ET, sub-tomogram averages were randomized between even/odd groups during refinement and resolution estimation (gold-standard FSC). Randomisation is not applicable for the budding yeast mutants generated in this study which were assayed against the WT strain.
Blinding	For cryo-ET, all data collection and image processing procedures were automatically performed in an unbiased manner which is generally used in the field of cryo-EM and cryo-ET.

Reporting for specific materials, systems and methods

We require information from authors about some types of materials, experimental systems and methods used in many studies. Here, indicate whether each material, system or method listed is relevant to your study. If you are not sure if a list item applies to your research, read the appropriate section before selecting a response.

Materials & experimental systems

n/a	Involved in the study
☒	☐ Antibodies
☐	☒ Eukaryotic cell lines
☒	☐ Palaeontology and archaeology
☒	☐ Animals and other organisms
☒	☐ Clinical data
☒	☐ Dual use research of concern
☒	☐ Plants

Methods

n/a	Involved in the study
☒	☐ ChIP-seq
☒	☐ Flow cytometry
☒	☐ MRI-based neuroimaging

Eukaryotic cell lines

Policy information about cell lines and Sex and Gender in Research

Cell line source(s)	S. cerevisiae strain NCYC74
Authentication	The S. cerevisiae strain was not authenticated
Mycoplasma contamination	The S. cerevisiae strain was not tested for mycoplasma contamination.
Commonly misidentified lines (See ICLAC register)	None

Plants

Seed stocks	*Report on the source of all seed stocks or other plant material used. If applicable, state the seed stock centre and catalogue number. If plant specimens were collected from the field, describe the collection location, date and sampling procedures.*
Novel plant genotypes	*Describe the methods by which all novel plant genotypes were produced. This includes those generated by transgenic approaches, gene editing, chemical/radiation-based mutagenesis and hybridization. For transgenic lines, describe the transformation method, the number of independent lines analyzed and the generation upon which experiments were performed. For gene-edited lines, describe the editor used, the endogenous sequence targeted for editing, the targeting guide RNA sequence (if applicable) and how the editor was applied.*
Authentication	*Describe any authentication procedures for each seed stock used or novel genotype generated. Describe any experiments used to assess the effect of a mutation and, where applicable, how potential secondary effects (e.g. second site T-DNA insertions, mosiacism, off-target gene editing) were examined.*
